# Physical activity, regulatory emotional self-efficacy, and suicidal ideation in adolescents: a variable-centered and person-centered approach

**DOI:** 10.3389/fpsyg.2026.1775139

**Published:** 2026-05-18

**Authors:** Mengxuan Liu

**Affiliations:** Jiangxi Institute of Technology, Nanchang, China

**Keywords:** adolescents, latent profile analysis, mediation analysis, mental health, physical activity, regulatory emotional self-efficacy, suicidal ideation

## Abstract

**Background:**

Suicidal ideation (SI) is an important early marker of suicide-related risk during adolescence. Clarifying modifiable factors associated with SI, as well as identifying adolescents with distinct patterns of SI, may help inform more targeted prevention efforts. This study investigated the relationships among physical activity (PA), regulatory emotional self-efficacy (RE), and SI, and further examined whether adolescents could be classified into latent SI subgroups.

**Methods:**

Adolescents were invited to complete self-report measures of PA, RE, and SI. Variable-centered analyses were used to test the associations among the study variables and the indirect association involving RE after adjusting for demographic covariates. A person-centered latent profile analysis (LPA) was then conducted to explore whether distinct SI profiles could be identified.

**Results:**

Higher PA was related to lower SI and higher RE. RE showed a significant indirect association between PA and SI. The LPA supported a two-profile solution, consisting of a high-SI profile with lower PA and weaker RE and a low-SI profile with higher PA and stronger RE.

**Conclusion:**

PA and RE were both associated with lower SI among adolescents and are relevant to prevention-oriented research and practice. The combined variable-centered and person-centered findings indicate that adolescent SI is better understood by considering both overall PA–RE–SI associations and subgroup differences in suicide-related cognition.

## 1 Introduction

Suicidal ideation (SI) refers to thoughts that range from wishing not to live to actively considering ending one’s life, and it is an important indicator of suicide-related risk during adolescence (Campisi et al., 2020; Orri et al., 2020; Uddin et al., 2019; Meter et al., 2023; Hu et al., 2026b). Worldwide epidemiological data identify suicide as a major source of mortality among adolescents aged 15–19 years, and population-based studies have documented 12-month SI in a considerable proportion of adolescents (Bertuccio et al., 2024; Hu et al., 2025b; Wasserman et al., 2021). Reports from UNICEF, WHO, and CDC also indicate that psychological distress, self-injury, and emotional difficulties have become increasingly prominent concerns among adolescents across different social and cultural contexts (Xu et al., 2025; Stevens, 2024; Carvajal-Velez et al., 2023). In China, survey-based studies have similarly documented a relatively high prevalence of SI among adolescents. However, suicide-related thoughts are often difficult to identify because stigma surrounding mental health problems and reluctance to disclose emotional distress may prevent adolescents from seeking help or reporting such experiences (Yan and Gai, 2022; Alqueza et al., 2023). If these difficulties are not recognized in time, they may affect academic functioning, peer relationships, and long-term developmental adjustment (Verboom et al., 2014; Nosè et al., 2025; Wang J. et al., 2025). Therefore, research on adolescent SI should not focus only on describing general risk status. Greater attention is needed to modifiable behavioral and psychological factors that can be incorporated into school- and community-based prevention programs (Bakken et al., 2025; Bakken et al., 2024). Compared with demographic characteristics or distal risk indicators, such factors may offer more feasible targets for early identification and intervention.

### 1.1 Physical activity and suicidal ideation

Physical activity (PA) represents a modifiable behavioral domain relevant to adolescent mental health. It can be promoted in school, family, and community contexts and is closely related to emotional functioning (Fu et al., 2025; Yuan et al., 2025; Li Z. et al., 2024). Adolescents who participate in PA more regularly tend to show fewer depressive symptoms, lower anxiety, and reduced perceived stress, which are all closely connected with SI (Wright et al., 2023; Hu et al., 2025c; Samsudin et al., 2024; Liu et al., 2025; Fabiano et al., 2023). PA may also be associated with lower rumination and catastrophic thinking, two cognitive processes that can intensify negative affect and maintain suicide-related thoughts (Chen et al., 2019; Luo et al., 2023; Ge et al., 2025; Wang et al., 2024). This association is particularly meaningful during adolescence, when emotion regulation abilities are still developing and affective responses to stress may be unstable (Ahmed et al., 2015; Guyer et al., 2016). However, evidence focusing specifically on PA and adolescent SI remains insufficient. In addition, the psychological resources that may help explain why PA is related to SI have not been fully clarified (Michael et al., 2020; Pfledderer et al., 2019).

### 1.2 The mediating role of regulatory emotional self-efficacy

Regulatory emotional self-efficacy (RE) denotes individuals’ perceived capacity for regulating negative emotional experiences (Alessandri et al., 2023; Zuffianò et al., 2025; Mesurado et al., 2018). From a conservation of resources perspective, RE may be understood as an internal psychological resource that contributes to adolescents’ emotional stability under stress (Hobfoll, 1989; Hobfoll, 2001; Reif et al., 2021). When adolescents have weaker RE, they may be more likely to view negative emotions as difficult to control, which can increase vulnerability to helplessness, emotional dysregulation, and SI (Liu et al., 2020; Colmenero-Navarrete et al., 2022). PA may be associated with RE because physical activity participation often involves goal pursuit, effort regulation, mastery experiences, peer interaction, and feedback from teachers or instructors (Zhang et al., 2024; Peng et al., 2025; Li X. et al., 2024). These experiences may gradually strengthen adolescents’ confidence in coping with emotional challenges. Therefore, RE may provide a psychological explanation for the association between PA and SI. However, this possible indirect association has not been sufficiently examined in large adolescent samples.

### 1.3 Latent profile analysis

SI may present differently across adolescents rather than following a uniform pattern. Some adolescents may report infrequent or mild suicide-related thoughts, whereas others may show more persistent or elevated ideation (Jobes et al., 2024; Hallensleben et al., 2024; Reeves et al., 2022). Importantly, adolescents with similar total SI scores may still differ in how they respond to specific SI items, suggesting that total-score approaches may overlook meaningful within-sample heterogeneity. Variable-centered analyses are useful for describing average associations among PA, RE, and SI, but they cannot determine whether adolescents can be grouped according to shared SI response patterns. LPA allows adolescents with comparable item-level SI responses to be grouped into latent profiles (Moore and Quartiroli, 2025). In the present study, LPA was applied to examine whether adolescents’ SI responses formed meaningful profiles and whether PA and RE varied across these profiles. This approach fits the study aims because PA and RE represent behavioral and psychological resources that may differ across adolescents with varying levels of suicide-related cognition (Díez-Gómez et al., 2020; Wei and Liu, 2024).

### 1.4 Current study

The present study examined PA, RE, and SI among adolescents by integrating variable-centered and person-centered analyses. The variable-centered analysis tested whether PA was associated with SI and whether RE showed a statistical indirect association in this relationship. The person-centered analysis used LPA to identify SI profiles and to compare PA and RE across the identified profiles. By combining these approaches, the study aimed to clarify both the overall PA–RE–SI associations and the heterogeneity of SI across adolescents. Guided by conservation of resources theory and existing empirical evidence, the study tested the following hypotheses:

*(H1)*: PA is negatively associated with SI.

*(H2)*: RE shows an indirect association in the relationship between PA and SI.

*(H3)*: Adolescents can be classified into distinct SI profiles that differ significantly in PA and RE.

## 2 Materials and methods

### 2.1 Participants

An *a priori* sample size estimation was conducted in G*Power 3.1 for the mediation analysis (Faul et al., 2007; Faul et al., 2009). Based on Cohen’s conventions, a small effect size was assumed (*f*^2^ = 0.02), with statistical power set to 0.95 and *α* set to 0.05. PA, RE, sex, and grade were entered as predictors. The required minimum sample size was approximately 550. The final analytic sample consisted of 3,319 adolescents, which exceeded the estimated requirement and was also sufficient for latent profile analysis (Wassertheil, 1970).

The survey was conducted from April to June 2025 in 12 secondary schools in a central province of China. A convenience sampling approach was adopted, and participating schools were included according to accessibility and willingness to cooperate with the study. As students in Grades 9 and 12 were preparing for major entrance examinations, only students in Grades 7, 8, 10, and 11 were invited. After school administrators granted permission, trained researchers administered the questionnaires during regular class periods. All students participated voluntarily, and written informed consent was obtained from both students and their guardians before data collection. The study procedures complied with the Declaration of Helsinki and were approved by the institutional ethics committee.

In total, 3,672 questionnaires were returned. Data screening was then conducted in three steps. First, two attention-check items, “select 1” and “select 3,” were included in the questionnaire, and participants who failed either item were excluded (*n* = 89). Second, questionnaires with identical responses across all items were removed (*n* = 121). Third, cases with missing or incomplete data on the main study variables were excluded through listwise deletion (*n* = 143). No imputation procedure was used. The final analytic sample therefore included 3,319 valid cases.

### 2.2 Measures

#### 2.2.1 Physical activity

PA was assessed with the Physical Activity Rating Scale-3 (PARS-3), revised by Liang in 1994 (Liang, 1994). The PARS-3 assesses physical activity engagement through three components: exercise intensity, exercise duration, and exercise frequency. Scores for intensity and frequency range from 1 to 5, and the duration score ranges from 0 to 4. The overall PA score is calculated as intensity × duration × frequency, producing a possible score range of 0–100. Higher scores represent greater participation in physical activity. Unlike psychological scales designed to measure a latent construct through multiple indicators, the PARS-3 is mainly a composite behavioral index that reflects the combined characteristics of exercise behavior. Its use is therefore supported by its scoring logic, previous application, and reliability evidence rather than by confirmatory factor analysis. The PARS-3 has been used frequently in studies of Chinese adolescents and young adults and has demonstrated acceptable measurement reliability in these populations (Huang et al., 2025a; Hu et al., 2025d; Huang et al., 2025b; Hu et al., 2026d). Previous work reported a Cronbach’s *α* of 0.713.

#### 2.2.2 Regulatory emotional self-efficacy

The Regulatory Emotional Self-Efficacy Scale (RES), originally developed by Caprara et al. (2008), was used to measure RE. The scale includes 12 items measuring adolescents’ perceived capability to express positive emotions, manage negative emotions, and regulate anger. Participants responded to each item on a 5-point Likert scale from 1 (not at all true of me) to 5 (very true of me). The item mean was used as the RES score, with higher values indicating stronger perceived emotional regulation capability. The RES was selected because previous research has conceptualized regulatory emotional self-efficacy as a multidimensional self-regulatory belief and has reported satisfactory psychometric performance for this measure in adolescent or Chinese samples (Chen and Liu, 2025; Wan et al., 2025; Tan et al., 2025). This evidence supports its use for assessing emotion-related self-efficacy rather than general emotional distress. The RES showed satisfactory reliability in the current sample, with Cronbach’s *α* = 0.864.

#### 2.2.3 Suicidal ideation

SI was evaluated using the Positive and Negative Suicide Ideation Scale (PANSI), a measure originally developed by Osman et al. and subsequently translated and validated in Chinese samples (Chen et al., 2021; Chang et al., 2009; Han et al., 2017). The PANSI consists of 14 items, including 6 positive-ideation items that were reverse-coded and 8 negative-ideation items (Brás et al., 2024). Participants reported the frequency of suicide-related thoughts during the previous 2 weeks using a 5-point Likert scale. Mean scores were used in the analyses, and higher scores reflected more severe SI. The PANSI was used because previous studies involving Chinese adolescent or youth samples have examined its measurement structure and reported evidence for its reliability and applicability in non-clinical samples (He et al., 2023; Zhang and Brown, 2007). The PANSI showed satisfactory reliability in the current sample, with Cronbach’s *α* = 0.899.

#### 2.2.4 Covariates

The mediation model adjusted for sex and grade. These variables were selected because they represent basic demographic and developmental characteristics that are closely related to adolescent SI. Prior research has shown that suicide-related thoughts may differ by sex, and grade level may capture developmental and school-stage differences in academic pressure, emotional maturity, and social adjustment (Zhang et al., 2019; Goldman-Mellor et al., 2018). Adjusting for sex and grade therefore helped reduce basic demographic confounding when estimating the associations among PA, RE, and SI. However, these covariates were not intended to provide exhaustive control for all potential confounders.

### 2.3 Data analysis

Statistical analyses were performed in SPSS 26.0 and Mplus 8.3. Descriptive statistics and Pearson correlation analyses were first conducted to summarize PA, RE, and SI and to examine their pairwise associations. Prior to the variable-centered analyses, PA, RE, and SI were transformed into standardized *z*-scores (*M* = 0, SD = 1). The mediation analysis was conducted in SPSS with Hayes’ PROCESS macro (Model 4) (Hayes et al., 2017; Hu et al., 2025a), while adjusting for sex and grade. Given the cross-sectional nature of the data, the model was used to estimate statistical indirect associations rather than causal mechanisms. Bootstrap estimation with 5,000 resamples was applied to generate 95% confidence intervals (CIs) for the indirect association. Because all variables were standardized before model estimation, the total, direct, and indirect effects shown in Table 1 are presented as standardized coefficients. Statistical significance for the indirect association was determined by whether the 95% bootstrap CI excluded zero.

**Table 1:** Bootstrap results for the indirect association.

Path	Effect	BootSE	BootLLCI	BootULCI	Relative proportion
Total effect	−0.387	0.016	−0.418	−0.357	100%
Direct effect	−0.071	0.015	−0.101	−0.041	18%
Indirect effect	−0.316	0.014	−0.344	−0.290	82%

For the person-centered analyses, latent profile models specifying one to five classes were fitted in Mplus with the 14 SI items entered as profile indicators. The SI items were treated as continuous variables, and robust maximum likelihood estimation (MLR) was used. To reduce the likelihood of local solutions, multiple random starts were specified and the number of final-stage optimizations was increased. The model solution associated with the highest log-likelihood value was retained. Profile selection considered statistical fit indices together with classification quality, profile size, and substantive interpretability. Following previous recommendations, each profile was required to include at least 5% of the total sample (Coelho et al., 2025). Lower values of AIC, BIC, and adjusted BIC (aBIC) were taken to indicate improved model fit (Vrieze, 2012). Significant LMR-LRT and BLRT results suggested that the k-class solution fit better than the *k* − 1 class solution, whereas entropy values closer to 1 indicated better classification precision. After the optimal profile model was identified, independent-samples *t*-tests were performed to compare PA and RE across the resulting profiles.

Several steps were used to reduce common method bias. The questionnaire was completed anonymously, all participants received standardized instructions, and reverse-coded items were included. As an initial assessment of common method bias, Harman’s single-factor test was performed. The variance explained by the first unrotated factor was 33.76%, which did not exceed the commonly applied 40% criterion (Podsakoff et al., 2003). This result suggested that no single factor dominated the covariance among the study measures. Nevertheless, Harman’s test offers only limited diagnostic evidence. Because PA, RE, and SI were all reported by the same participants at the same time point, shared method variance cannot be ruled out and should be considered when interpreting the strength of the observed associations.

## 3 Results

### 3.1 Preliminary analyses

Table 2 summarizes the means, standard deviations, and Pearson correlations among PA, RE, and SI. The possible score ranges were 0–100 for PA and 1–5 for both RE and SI. PA showed considerable variability, which may be related to the multiplicative scoring method of the PARS-3, where exercise intensity, duration, and frequency are combined into a composite score. The mean scores were 30.837 for PA (SD = 29.660), 3.214 for RE (SD = 0.886), and 2.005 for SI (SD = 0.632). PA was positively correlated with RE (*r* = 0.546, *p* < 0.01) and negatively correlated with SI (*r* = −0.413, *p* < 0.01). RE was also negatively correlated with SI (*r* = −0.663, *p* < 0.01). These correlations indicate that adolescents with higher PA and stronger RE tended to report lower levels of SI.

**Table 2:** Descriptive statistics and correlations among study variables (*N* = 3,319).

Variable	*M*	SD	Skewness	Kurtosis	Possible range	1	2	3
1. PA	30.837	29.660	0.737	−0.672	0–100	1		
2. RE	3.214	0.886	−0.200	−0.878	1–5	0.546^**^	1	
3. SI	2.005	0.632	1.699	3.429	1–5	−0.413^**^	−0.663^**^	1

PA, physical activity; RE, regulatory emotional self-efficacy; SI, suicidal ideation. Possible score ranges were PA = 0–100, RE = 1–5, and SI = 1–5. Higher scores indicate higher levels of the corresponding constructs. **p < 0.01.

### 3.2 Variable-centered analysis

A mediation model was estimated to examine whether RE was statistically involved in the association between PA and SI, with sex and grade included as covariates. As shown in Table 3, PA was positively associated with RE (*β* = 0.529, *t* = 36.435, *p* < 0.001). Given that the continuous variables were standardized before analysis, this coefficient reflects a moderate-to-large association, indicating that adolescents with higher PA scores also tended to report stronger perceived emotional regulatory capability.

**Table 3:** Results of the mediation model.

Predictor/model statistic	RE		SI	
	*β*	*t*	*β*	*t*
Sex	−0.249	−8.497^***^	0.242	9.219^***^
Grade	0.035	2.652^**^	−0.067	−5.732^***^
PA	0.529	36.435^***^	−0.071	−4.648^***^
RE			−0.598	−38.885^***^
*R* ^2^	0.315		0.463	
*F*	508.102^***^		715.125^***^	

PA, physical activity; RE, regulatory emotional self-efficacy; SI, suicidal ideation. Sex and grade were included as covariates. **p < 0.01, ***p < 0.001.

**Figure 1:**
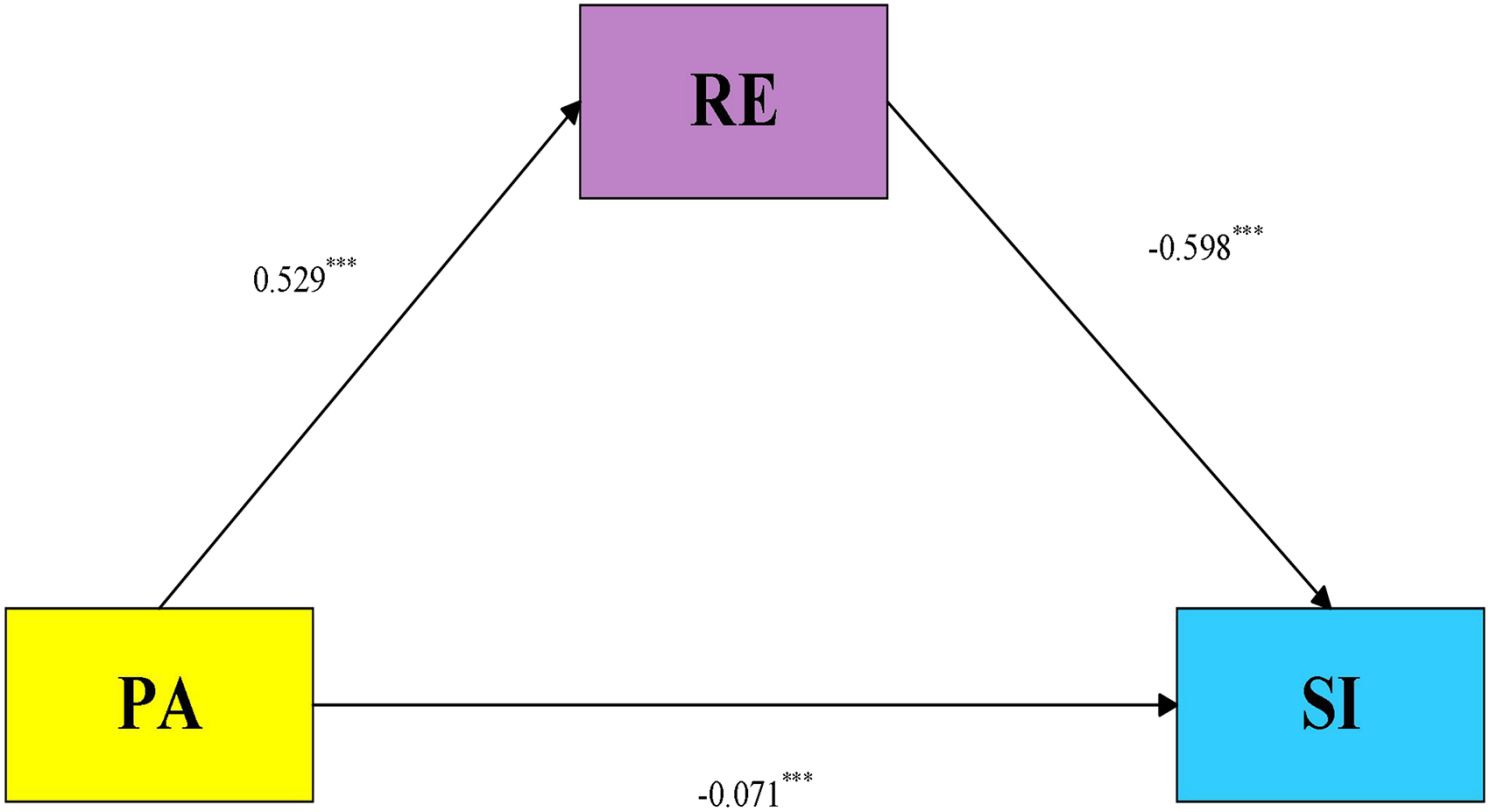
Mediation model linking PA, RE, and SI. PA, physical activity; RE, regulatory emotional self-efficacy; SI, suicidal ideation. ^***^p < 0.001.

In the model predicting SI, PA remained negatively associated with SI after RE was entered, although the standardized coefficient was relatively small (*β* = −0.071, *t* = −4.648, *p* < 0.001). By comparison, RE showed a much larger negative association with SI (*β* = −0.598, *t* = −38.885, *p* < 0.001). In the adjusted model, the association between RE and SI appeared stronger than that observed for PA and SI. The model accounted for 31.5% of the variance in RE (*R*^2^ = 0.315) and 46.3% of the variance in SI (*R*^2^ = 0.463), both *p* < 0.001. Thus, the results point to a considerable explanatory capacity of the model, particularly for SI, while also showing that the direct PA–SI association was markedly reduced when RE was considered. Sex and grade were also significantly associated with SI, suggesting that demographic differences should be considered when interpreting adolescents’ SI (see Figure 1).

The indirect association was evaluated using 5,000 bootstrap resamples. As reported in Table 1, the total association between PA and SI was significant (effect = −0.387, 95% CI [−0.418, −0.357]). When RE was included in the model, the direct association between PA and SI remained significant but was relatively small (effect = −0.071, 95% CI [−0.101, −0.041]). The bootstrap confidence interval for the indirect association through RE did not include zero (effect = −0.316, 95% CI [−0.344, −0.290]), indicating a significant indirect association. The indirect association accounted for approximately 82% of the total effect. This proportion indicates that the PA–SI association was substantially attenuated after RE was included in the model; however, it should be interpreted as a descriptive decomposition of standardized associations rather than as evidence that RE represents the only or dominant causal mechanism.

### 3.3 Person-centered analysis

Using the 14 SI items as indicators, latent profile solutions ranging from one to five classes were estimated. The model-fit results are reported in Table 4. As additional profiles were added, AIC, BIC, and aBIC values decreased, suggesting improved numerical fit in models with more profiles. However, the final profile solution was not determined solely by information criteria. We also considered whether the profiles were sufficiently large, whether classification quality was acceptable, whether the item-level response patterns were distinguishable, and whether the solution could be substantively interpreted.

**Table 4:** Fit indices for latent profile models based on SI.

Profiles	AIC	BIC	aBIC	LMR-LRT	BLRT	Entropy	1	2	3	4	5
1	128225.549	128396.557	128307.589	N/A	N/A	1	100.00				
2	114522.593	114785.212	114648.582	<0.001	<0.001	0.996	8.56	91.44			
3	110597.966	110952.196	110767.904	<0.001	<0.001	0.862	52.03	40.74	7.23		
4	107788.547	108234.389	108002.435	<0.001	<0.001	0.894	47.36	3.31	42.42	6.91	
5	105916.322	106453.775	106174.159	<0.001	<0.001	0.913	47.26	3.59	42.48	3.15	3.52

The two-profile model showed a clear and interpretable distinction between adolescents with generally low SI responses and those with consistently elevated SI responses. The Lo–Mendell–Rubin adjusted likelihood ratio test and the bootstrap likelihood ratio test both reached statistical significance (*p* < 0.001), indicating that the two-profile model improved upon the one-profile model. The entropy value was 0.996, suggesting high classification precision. The smaller profile represented 8.56% of the sample, which exceeded the recommended 5% minimum and therefore did not indicate an extremely small or unstable subgroup.

Although the three-profile model and models with more profiles showed lower information criteria, the additional profiles did not provide a sufficiently distinct or theoretically meaningful pattern beyond the main low-SI versus high-SI distinction. Some higher-order solutions also produced smaller profiles, which reduced their practical interpretability. For these reasons, the two-profile solution was retained as the most parsimonious and interpretable representation of the dominant SI response pattern in this sample. This decision should be understood as an empirical modeling choice rather than as evidence that adolescent SI has only two fixed categories.

**Figure 2:**
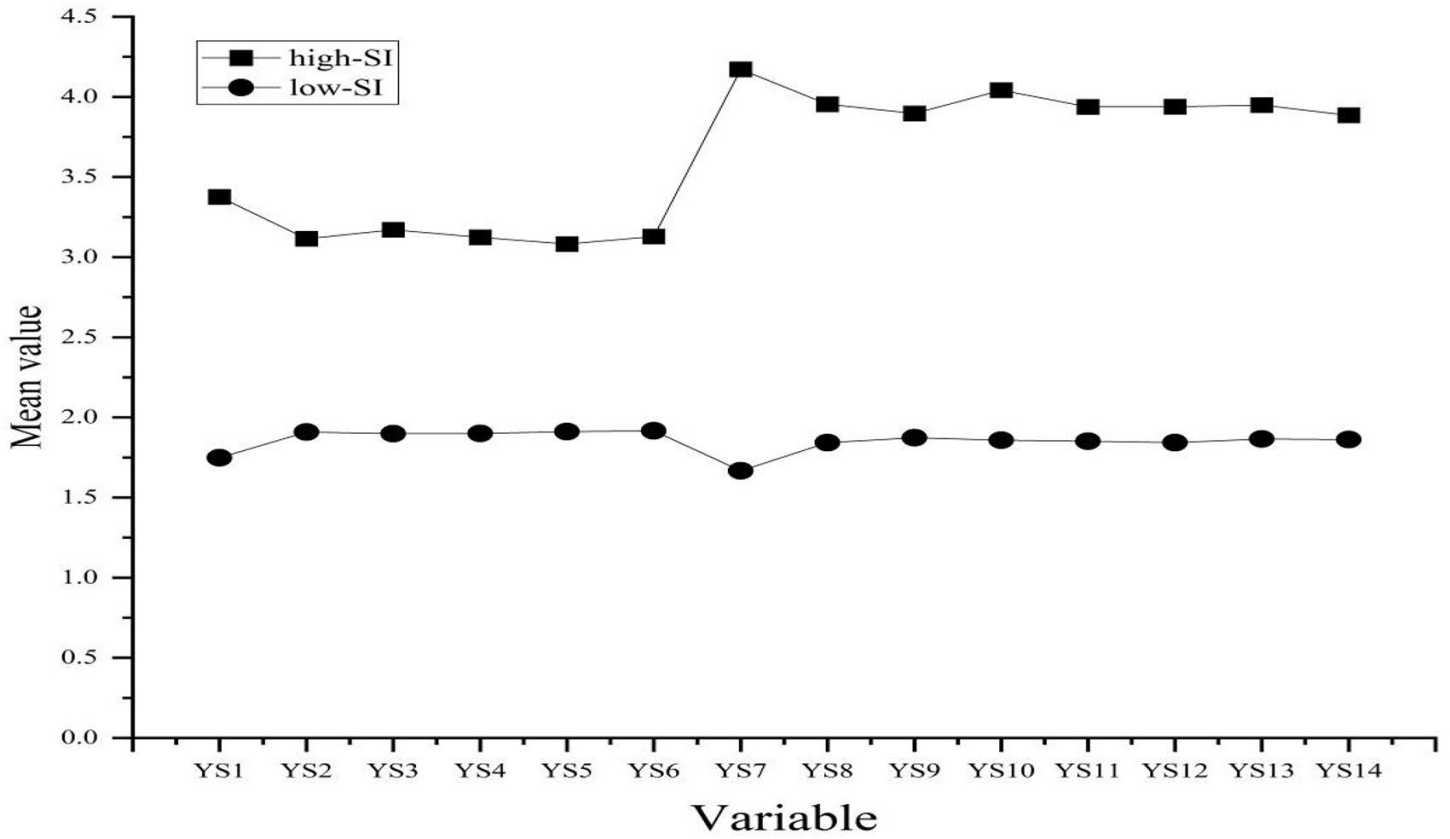
Item-level response patterns of the two SI profiles.

Figure 2 displays the item-level response patterns of the two profiles. The low-SI profile included 3,035 adolescents, representing 91.44% of the sample, and was characterized by consistently low scores across the SI items. The high-SI profile included 284 adolescents, representing 8.56% of the sample, and showed higher scores across the same items. These item-level patterns indicated a clear separation between the two profiles.

Table 5 presents the comparisons of PA and RE between the two profiles. Adolescents in the low-SI profile reported higher PA and RE than those in the high-SI profile. These differences were statistically significant for both PA (*t* = −14.023, *p* < 0.001) and RE (*t* = −38.435, *p* < 0.001).

**Table 5:** Differences in PA and RE between the two SI profiles.

Variable	Profile	*N*	*M*	SD	*t*
PA	High-SI	284	12.26	22.67	−14.023^***^
	Low-SI	3,035	32.58	29.64	
RE	High-SI	284	1.99	0.53	−38.435^***^
	Low-SI	3,035	3.33	0.82	

PA, physical activity; RE, regulatory emotional self-efficacy; SI, suicidal ideation. ***p < 0.001.

## 4 Discussion

### 4.1 Overview of the main findings

This study investigated the associations among PA, RE, and SI in adolescents using both variable-centered and person-centered analyses. Three key findings were observed. First, adolescents with higher PA tended to report lower SI. Second, RE showed a significant indirect association between PA and SI, indicating that adolescents’ confidence in regulating negative emotions may be relevant to this association. Third, LPA identified two SI profiles, suggesting that suicide-related cognition differs across adolescents rather than being evenly distributed in the sample. Viewed through conservation of resources theory, PA represents a behavioral form of resource, whereas RE reflects an internal self-regulatory resource that is relevant to adolescents’ emotional adjustment in stressful contexts. Overall, the findings indicate that both behavioral engagement and emotion-related self-beliefs are associated with variation in adolescent SI.

### 4.2 Associations between physical activity and suicidal ideation

In line with Hypothesis 1, PA showed a negative association with SI. Adolescents reporting higher levels of PA tended to report fewer suicide-related thoughts. A possible explanation is that PA is closely related to emotional functioning. Previous studies have linked regular PA with fewer depressive and anxiety symptoms, reduced stress reactivity, and better sleep quality, all of which are relevant to SI (Singh et al., 2023; Samsudin et al., 2024; Recchia et al., 2023). PA may also be related to lower levels of maladaptive cognitive processes, including rumination and catastrophic thinking, which can increase vulnerability to negative emotional states (Wang S. et al., 2025; Zhou and Wang, 2025; Lu et al., 2024). Therefore, PA may serve as a modifiable behavioral factor associated with lower SI in adolescence. This finding also supports the value of providing adolescents with accessible and sustainable opportunities for PA in school and family settings.

### 4.3 The role of regulatory emotional self-efficacy in the PA–SI association

Consistent with Hypothesis 2, the analysis indicated a significant statistical indirect association involving RE in the PA–SI relationship. In the mediation model, PA was moderately and positively associated with RE, whereas RE showed a relatively strong negative association with SI. After RE was included in the model, the direct association between PA and SI became small but remained statistically significant. This pattern suggests that adolescents’ perceived emotional regulatory capability is closely involved in the PA–SI association.

This finding is theoretically meaningful. PA often provides adolescents with repeated experiences of goal-directed effort, mastery, peer interaction, and feedback from teachers or instructors (Yang et al., 2026; Hu et al., 2026c; Xiang et al., 2026). These experiences may help adolescents develop stronger confidence in managing negative emotions and coping with stress. Adolescents with stronger RE may be less likely to perceive negative affect as uncontrollable, enduring, or overwhelming. From this perspective, RE can be understood as a proximal psychological resource that is more directly related to suicide-related cognition than PA, which represents a broader behavioral factor (Hu et al., 2026a; Chen et al., 2026).

The magnitude of the indirect association warrants a cautious interpretation. In the present model, the indirect effect represented approximately 82% of the total PA–SI association. Statistically, this proportion indicates that the PA–SI association was substantially attenuated after RE was entered into the model. It should not be interpreted as evidence that RE is the only or dominant causal mechanism linking PA with SI. Several methodological and conceptual factors may have contributed to this large estimate. First, RE and SI are both closely related to adolescents’ emotional functioning, and this conceptual proximity may partly explain the strong association between them. Second, PA, RE, and SI were all measured using self-report questionnaires administered in the same survey setting, which may have introduced shared method variance and thereby partly contributed to the magnitude of the observed associations. Third, because the data were cross-sectional, the temporal order among PA, RE, and SI cannot be established.

Therefore, the indirect association should be regarded as a theoretically informative statistical pattern rather than evidence of a confirmed causal pathway. The findings highlight the relevance of RE in understanding the association between PA and SI, but they also indicate the need for more rigorous research designs. Future longitudinal and intervention studies should examine whether higher PA is followed by later improvements in RE and subsequent reductions in SI. Such studies should also include multi-informant, behavioral, or physiological measures to clarify whether the observed indirect association reflects a stable psychological process rather than measurement overlap, shared method variance, or unmeasured emotional distress.

### 4.4 Heterogeneity in suicidal ideation profiles

Consistent with Hypothesis 3, the LPA identified two SI profiles. The larger profile was characterized by consistently low SI responses, whereas the smaller profile showed elevated responses across the SI items. This finding indicates that suicide-related cognition was not evenly distributed across adolescents. Instead, a smaller subgroup showed a more concentrated pattern of elevated SI, which may be particularly relevant for prevention-oriented screening and support.

The selection of the two-profile solution should be interpreted carefully. Although models with additional profiles produced lower AIC, BIC, and aBIC values, they also generated smaller subgroups and less clearly interpretable response patterns. The two-profile solution provided a clearer and more parsimonious representation of the main distinction observed in the data: adolescents with generally low SI and adolescents with consistently higher SI. The high entropy value suggested strong classification precision, but entropy alone was not treated as the basis for model selection. The final decision was made by considering statistical fit, profile size, item-level response patterns, and theoretical interpretability (Chen B. et al., 2025).

At the same time, the two-profile solution should not be taken to mean that adolescent SI has only two natural forms. SI is a complex and dynamic phenomenon that may vary in intensity, duration, cognitive content, emotional context, and developmental course. The present model captured the dominant pattern in this sample, but more complex profile structures may emerge in other populations or when additional indicators are included (Xiang et al., 2025). For example, future studies may identify profiles characterized by passive death wishes, active suicidal thoughts, fluctuating ideation, or co-occurring emotional distress. Longitudinal person-centered analyses would be especially useful for examining whether adolescents remain in the same SI profile or move between profiles over time.

The profile comparisons further showed that adolescents in the high-SI profile reported lower PA and weaker RE than those in the low-SI profile. This pattern suggests that behavioral and psychological resources may differ across adolescents with different SI response patterns. Drawing on conservation of resources theory, adolescents with fewer opportunities for PA and weaker confidence in regulating negative emotions may have fewer resources for managing stress and affective difficulties. However, because profile differences were examined cross-sectionally, these results should be interpreted as descriptive group differences rather than evidence that low PA or weak RE determines profile membership. Future research should examine whether contextual factors, such as school climate, family support, peer relationships, and access to PA resources, may help identify adolescents with a greater likelihood of belonging to the high-SI profile.

### 4.5 Practical implications for prevention and intervention

The results are relevant to school- and community-based efforts to promote adolescent mental health and prevent suicide-related risk. First, the identification of low-SI and high-SI profiles supports the need for prevention strategies that account for subgroup differences. Adolescents in the low-SI profile may be appropriate for universal programs that maintain regular PA and strengthen existing emotional resources. In contrast, adolescents in the high-SI profile require closer attention and more structured support that combines PA promotion with emotional regulation training. Second, the strong association between RE and SI highlights the importance of emotion-related self-regulatory beliefs in prevention-oriented work. Physical education and school-based health programs should not focus only on increasing the amount of PA, but also on creating experiences that strengthen adolescents’ confidence in managing negative emotions. Activities involving mastery experiences, supportive feedback, cooperative participation, and guided reflection may be especially useful for building this confidence (Hu et al., 2025b,c; Yue et al., 2025). Finally, the results point to the importance of supportive environments across schools, families, and communities. Sustainable access to PA opportunities, emotionally supportive relationships, and timely identification of adolescents with elevated SI profiles are all relevant components of prevention. Thus, the present findings support an integrated prevention approach that combines behavioral engagement, emotional regulation support, and subgroup-sensitive screening (Zhang et al., 2026; Chen X. et al., 2025).

### 4.6 Contributions of the present study

This study adds to the existing literature in several respects. First, it broadens current research on adolescent SI by considering PA as a modifiable behavioral factor together with RE as an emotion-related psychological resource within a unified analytic framework. Previous studies have often examined behavioral or psychological correlates of SI separately, whereas the present study considered how these two types of resources were jointly associated with suicide-related cognition. Second, the study provides a more differentiated account of the PA–SI association by showing that RE was strongly involved in this relationship. Rather than treating PA as a uniformly protective behavior, the findings suggest that adolescents’ perceived capacity to regulate negative emotions is an important psychological context in which the PA–SI association should be understood. This contribution is particularly relevant for prevention research, because it points to the value of combining PA promotion with efforts to strengthen emotion-regulation beliefs. Third, the use of LPA adds person-centered evidence to the variable-centered findings.

The identification of low-SI and high-SI profiles indicates that suicide-related cognition was not evenly distributed across adolescents. Moreover, the profile comparisons showed that adolescents in the high-SI profile reported lower PA and weaker RE. These findings help connect overall statistical associations with subgroup heterogeneity and provide a basis for more stratified prevention strategies. Finally, the study highlights the need to consider behavioral engagement and psychological resources together when examining adolescent SI. Although the data do not allow causal inference, the integration of mediation analysis and latent profile analysis offers complementary evidence: one approach describes associations among variables, whereas the other identifies adolescents with distinct SI response patterns. This combined perspective supports a more differentiated approach to adolescent SI research by linking average-level associations with subgroup-specific patterns of risk.

### 4.7 Limitations and directions for future research

Several limitations should be acknowledged when interpreting the findings. First, the participants were drawn from 12 schools within one geographic region, and the schools were selected mainly on the basis of accessibility and willingness to participate. This recruitment strategy may have led to selection bias and may restrict the extent to which the findings can be generalized to broader adolescent populations. In addition, because the response rate was not formally documented, the representativeness of the analytic sample cannot be fully assessed. Future studies should employ more systematic sampling strategies and include adolescents from a wider range of regional and cultural backgrounds.

Second, the cross-sectional nature of the data limits causal interpretation. Although the mediation analysis showed a significant indirect association, it cannot establish the temporal sequence among PA, RE, and SI. The results should therefore be understood as statistical associations rather than evidence of causal pathways. Longitudinal and experimental studies are needed to determine whether PA is followed by later changes in RE and SI.

Third, PA, RE, and SI were all measured by self-report within a single survey administration. This measurement approach may have increased the risk of response bias and shared method variance, because the three variables were reported by the same participants at the same time point. Although anonymous responding, standardized instructions, reverse-coded items, and Harman’s single-factor test were used to reduce and evaluate common method bias, these procedures cannot fully remove this possibility. Harman’s test should therefore be regarded as a preliminary diagnostic procedure rather than conclusive evidence that common method bias was absent. For this reason, the strong association between RE and SI, as well as the large indirect effect in the mediation model, should be interpreted with caution.

Fourth, although the instruments used in this study were selected with reference to prior psychometric evidence in Chinese adolescent or youth samples, their factor structures were not re-examined in the present dataset. This limitation is relevant to both the mediation analysis and the latent profile analysis, because measurement properties may vary across sex, grade, or risk subgroups. Future studies should further test factor structure and measurement invariance before drawing stronger conclusions about subgroup differences or latent SI profiles. Combining self-report data with parent or teacher reports, behavioral assessments, or physiological indicators would also help reduce reliance on a single measurement source.

Finally, the covariates included in the analyses were limited. Sex and grade were controlled because they are basic demographic and developmental indicators, but other factors that may shape adolescent SI were not measured. These include family functioning, peer relationships, socioeconomic conditions, school climate, depressive and anxiety symptoms, and sleep quality. The absence of these variables leaves open the possibility of residual confounding and may have affected the estimated associations among PA, RE, and SI. In addition, profile differences were examined using independent-samples t-tests, which allowed direct comparisons between the high-SI and low-SI groups but did not identify factors associated with profile membership. Future work should incorporate a broader set of contextual and psychological variables and use approaches such as multinomial logistic regression to examine predictors of membership in the high-SI profile.

## 5 Conclusion

This study integrated variable-centered and person-centered analyses to investigate the relationships among PA, RE, and SI in adolescents. Higher PA was associated with lower SI, and RE showed a significant indirect association in this relationship. LPA further identified low-SI and high-SI profiles, with adolescents in the low-SI profile reporting higher PA and stronger RE than those in the high-SI profile. These findings suggest that adolescent SI is related not only to overall levels of behavioral engagement and emotion-related self-regulatory beliefs, but also to subgroup differences in SI response patterns. Although causal inference is not warranted, the results support prevention-oriented strategies that promote PA, strengthen emotion-regulation beliefs, and attend to adolescents with elevated SI profiles.

## Data Availability

The raw data supporting the conclusions of this article will be made available by the authors, without undue reservation.
